# The everyday use of assistive technology by people with dementia and their family carers: a qualitative study

**DOI:** 10.1186/s12877-015-0091-3

**Published:** 2015-07-24

**Authors:** Grant Gibson, Claire Dickinson, Katie Brittain, Louise Robinson

**Affiliations:** School of Applied Social Sciences, Colin Bell Building, University of Stirling, Stirling, FK9 4LA Scotland, UK; Institute of Health & Society, Newcastle Biomedical Research Building, Newcastle University, Campus for Ageing and Vitality, Newcastle upon Tyne, NE4 5PL UK

**Keywords:** Assistive technology, Dementia, Carers, Social care

## Abstract

**Background:**

Assistive Technology (AT) has been suggested as a means by which people with dementia can be helped to live independently, while also leading to greater efficiencies in care. However little is known about how AT is being used by people with dementia and their carers in their daily routines. This paper reports on a qualitative study exploring the everyday use of AT by people with dementia and their families.

**Methods:**

The research employed a qualitative methodology. Semi structured interviews took place with 39 participants, 13 people with dementia and 26 carers. Key themes were identified using thematic analysis and the constant comparative method.

**Results:**

Three categories of AT use in everyday settings were identified; formal AT, accessed via social care services, ‘off the shelf AT’ purchased privately, and ‘do it yourself’ AT, everyday household products adapted by families to fulfil individual need in the absence of specific devices. Access to AT was driven by carers, with the majority of benefits being experienced by carers. Barriers to use included perceptions about AT cost; dilemmas about the best time to use AT; and a lack of information and support from formal health and social care services about how to access AT, where to source it and when and how it can be used.

**Conclusions:**

It has been argued that the ‘mixed economy’ landscape, with private AT provision supplementing state provision of AT, is a key feature for the mainstreaming of AT services. Our data suggests that such a mixed economy is indeed taking place, with more participants using ‘off the shelf’ and ‘DIY’ AT purchased privately rather than via health and social care services. However this system has largely arisen due to an inability of formal care services to meet client needs. Such findings therefore raise questions about just who AT in its current provision is working for and whether a mixed market approach is the most appropriate provider model. Everyday technologies play an important role in supporting families with dementia to continue caring; further research is needed however to determine the most effective and person-centred models for future AT provision.

## Background

As populations’ age, greater numbers of people are living with age-related conditions such as dementia; by 2050, it is estimated that 135 million people around the world will live with dementia [1]. Of all chronic diseases, dementia is one of the most important contributors to dependence and disability and costs to society [2, 3]. In 2010, the global cost of dementia care was estimated at US $604 billion and predicted to rise to US $1 trillion by 2030 [2, 3]. With such demands on care costs increasing attention is being paid to improving not only the quality but also the efficiency of service provision. Assistive technology (AT) has been identified as one area in which both improvements and possible cost savings, in care delivery can be made [4, 5]. The term assistive technology incorporates a wide range of devices, ranging from simple, low-cost devices to complex home monitoring systems using electronic information and communication technology (ICT). In addition, technology use in everyday dementia care also includes the use of a range of everyday or household devices and objects, which will not be defined as ‘AT’ or even as ‘technology’ but which are routinely used to assist with care functions and household tasks [6, 7]. Examples include using labour saving devices such as kitchen equipment to make everyday tasks easier, or even the use of simple noteboards, signs or labels to help identify rooms, or to provide instruction about how to use objects. Despite relatively limited evidence for the effectiveness of AT in chronic illness management [8–10], particularly in dementia care [11], UK national policy continues to focus on the ‘mainstreaming’ of AT, into routine care and the delivery of AT to scale, commonly in the form of ‘telecare’ with the aim of promoting safety, security and enabling independence in the form of autonomous living [12, 13].

A key part of the mainstreaming of AT is the development of a ‘mixed economy’ approach to service provision, with access taking place through both state funded social care and private provision [6, 13, 14]. Numerous developments have taken place to facilitate the growth of a mixed AT market including access to AT through charitable organisations and web-based information resources, large scale government initiatives to promote AT and the provision funding to facilitate implementation of AT services [13, 15, 16]. However barriers to the widespread dissemination of AT persist demonstrated in the UK by the failure of a recent attempt to introduce a mass market telecare product via commercial retail stores; low awareness amongst potential users and the general public was suggested as a possible reason for this [17]. The successful implementation of AT in everyday care has been shown to be influenced by configurations of both AT and general technologies, with these configurations being tailored to individual need [18, 19]. Yet how these configurations of AT, are used when giving and receiving dementia care, including their use in conjunction with general household or other technologies has seen little empirical study.

To date, research on AT in dementia has focused on small scale AT development or the ethical issues surrounding AT use (e.g. GPS technologies) [11, 20–26]. Although a large scale randomised controlled trial to test the effectiveness and cost effectiveness of AT in dementia care is now underway in the UK [27], there has been limited research to date exploring how people with dementia and their families use technology in their daily lives [7, 28]. The aim of this paper is to explore how people with dementia and their family carers use AT in their everyday lives, identify the types and range of devices they use, and the issues which influenced technology adoption within their usual care routines.

## Methods

The objective of this research was to explore levels of awareness and usage of technology, use including AT, among people with dementia and their family carers. Thirty-nine semi structured interviews were carried out between September 2013 and November 2014 with a purposive sample of 13 people dementia, 18 current family carers and 8 former carers. Inclusion criteria for people with dementia included: a formal diagnosis of dementia of any type; the capacity to give formal consent to participate in the study, and to be living independently at home, either alone or with an informal carer. The inclusion criteria for informal carers was individuals who were currently or had previously provided informal care for a person with a formal dementia diagnosis. In five cases, carers of an individual who lacked capacity to give informed consent volunteered to participate in the study; these carers were interviewed individually. In most cases carers were spousal partners or daughters. All people with dementia were living at home, with 11 living with a carer and 2 living alone. All participants were using general household technologies, either as part of daily life or to explicitly assist with care. In addition 21 participants (7 PwD and 14 carers) also had experiencing of using AT in some form. Participants were aged between 49–92 years: (people with dementia age range 49–91, mean age 72; carer age range 49–82, mean age 61). Participants were recruited from settings in the North East of England including local dementia cafes, a day centre/supported living service, a local authority telecare service, a GP surgery and a local forum promoting public participation in research. Interviews took place in a location convenient to the participant. In the majority of cases this was their own home (n = 30), via telephone (n = 1) or in a local dementia cafe (n = 1). In addition several interviews with former carers took place in their workplaces (n = 7). Ethical approval for this study was given by East of England Research Ethics Committee (ref 11/EE/0505).

Where possible people with dementia and their carers were interviewed individually. One interview was conducted with the person with dementia, with a further interview being carried out with informal carers by a second interviewer at the same time. This process ensured that views and awareness regarding AT among carers and people with dementia, and any differences between them could be explored separately. People with dementia were given the opportunity to be interviewed with their carers if they preferred; however in all but two cases they were happy to be interviewed separately. Interview schedules exploring access and use of AT by people with dementia and their carers were developed following a literature review [6, 29]. For those unfamiliar with AT, and to illustrate the scope and types of AT products currently available, participants were also shown photographs of AT devices. Photographs of devices included images of community alarms and telecare, Global Positioning System (GPS) location monitoring devices, signage, reminiscence tools and dementia friendly furniture. Interviews sought to further explore levels of awareness about AT, and to explore people’s experiences of AT products they were using or had used in the past (see box 1).

**Table:** 

Box 1. Topic areas explored within individual participant interviews	
•	General feelings about the use of technology to assist people with dementia
•	Views about specially designed AT used in dementia care (via demonstration of photographs)
•	Current use or non-use of everyday technology and specially developed AT
•	Experiences of accessing both information about AT and specific devices
•	Views regarding the use of or non-use of AT as the dementia progresses

Interviews with people with dementia lasted between 10 min and 1 h, with an average time of 30 min. Interviews with carers took approximately 1 h, with a range of 45 min to 2.5 h. Data was analysed using thematic analysis, with analysis also drawing upon the constant comparative method [30]. Interviews were initially coded by the first author, with initial themes being developed into a coding framework. This framework was then discussed with other researchers on the project, which in turn informed further data collection, and the revision of earlier themes. Results regarding the everyday use of AT, and issues influencing this usage are reported in this paper.

## Results

### What types of AT are used by people with dementia and their family carers?

We identified three main categories of AT use in everyday settings:

- ‘Formal’ AT: devices accessed via health and social care services following a ‘professional’ needs assessment
- Off the shelf’ AT: devices purchased by families directly from the private sector and
- ‘Do it yourself’ AT: everyday devices or systems developed, utilised or adapted by families themselves.

‘Formal’ AT comprised devices designed by AT manufacturers to facilitate assisted living activities, and which were predominantly supplied by social care services. Example products included community alarms, telecare and GPS location monitoring equipment. While this category of AT has received greatest policy attention, only 5 people with dementia accessed AT via this route. Formal AT were usually provided by local authority community alarm/telecare services, often after complex referral or assessment processes.

*I can remember when this assessor woman came out. And she was very nice, but it was all very much like there are sheets and sheets of tick boxes (…). It was like “Well, if you score on this one, you know, you’ll get this, if you score on that one you’ll get that, and how much money have you got,” (…) Very impersonal I thought and not very nice for my mum either. (Carer; C114)*

The second category was ‘off the shelf’ AT, dementia-friendly versions of existing devices; examples in this group included dementia-friendly signage, clocks, telephones, remote controls or GPS location monitors. An important feature of ‘off the shelf’ AT devices was that they were purchased directly from AT manufacturers, dementia/disability charities or from voluntary organisations, usually following a carers’ own research or after receiving recommendations from family, friends or other people living with dementia;

*That’s (the day clock) new, that’s recently just – it hasn’t been out long, it said it on the website. The write-ups on it were really good, and the feedback, you know, people’s feedback was brilliant on it (Carer; C104).*

Finally, the most common devices used by participants were ‘do it yourself’ AT, in which everyday household technologies were used to fulfil AT functions. Groups of AT in this category included: commercially available devices such as telephones, remote controls or kitchen appliances; simple devices such as whiteboards or post-it notes and the creation of individual ‘AT solutions’ using readily available information and communication technologies. Specific examples included two cases of buying a commercial GPS monitoring device, and two cases in which carers connected Closed Circuit Television (CCTV) cameras to a PC, tablet computer or smartphone via a home Wi-Fi network. Do it yourself AT could be simple or complex, were usually used in the absence of any formal AT equivalents, and were purchased from commercial retail sources such as online retailers, catalogues or high street stores.

*Interviewer: Have you ever seen anything like that (simple mobile phone), do you think you would know where to go to buy something like that if you were looking for one?*

*Person with dementia (P112): Where to buy? X (a UK electrical goods store) I think. Our phone was at Y (a UK store selling a range of household, leisure and electrical products), and I specifically went to them because they, they were on- making a special offer, you know, and it sounded great.*

A second feature of ‘do it yourself’ AT was modifying and adapting existing household products to make them easier for people with dementia to use, or using existing technologies in novel ways. In the example below, the family carer used a novelty motion sensor shaped like a cockerel to alert him if his wife approached the front door.

*I‘ve put an alarm on the door since I took her key off her, I lock up at night anyway, but what I do, I’ve got a cockerel, plastic cockerel, I used to stand on the thing and as you walk past it used to crow (Laughter). If I was upstairs I knew she was heading for the front door. (Carer; C107).*

Such devices were used in place of formal AT, either because carers did not know about their AT equivalents or how to access them, or because carers preferred to develop their own alternatives to AT using everyday technologies.

### How is AT used by people with dementia and their family carers?

Accounts of AT use predominantly focused on the benefits AT brought to carers and their caring roles. For example, telecare or GPS monitors were used by carers to monitor the movements and activities of a person with dementia in order to reduce the physical and emotional burdens of care. One carer installed a home-made AT system using CCTV cameras, a smartphone and a home Wi-Fi network. By enabling him to monitor his wife remotely, this system gave him/her a limited degree of freedom and independence:

*It means I used to be able to have a bit of a life outside of (wife), you know. I still had my freedom that I could away know that she was in the house and I could view her at any time. I could stand anywhere in the world if I wanted to and watch her. (Carer; C106).*

AT were only rarely used to assist people with dementia with their day to day, household or leisure tasks. Where such devices were used, they were usually ‘do it yourself’ (DIY); household labour saving devices or simplified, commercial versions of electronic household products such as landline or mobile telephones;

*My mum’s got (a mobile phone) and I have difficulty with that ‘cause all her mobile phones were like tiny little buttons. So I had seen a Doro (simple phone manufacturer), I don’t know if you’ve heard of the Doro ones? (…). So I got her one of those, so she’s got one with big buttons on. You need something very, very simple. (Carer; C109).*

Formal AT, such as GPS or telecare, could be introduced without the person with dementia necessarily understanding or remembering their purpose, or even knowing they had been installed. While most people with dementia knew that technologies such as GPS monitors or pendant alarms had been introduced to help them, several did not understand their purpose or had forgotten how they worked.

*You give her something quite simple and it just doesn’t sink in how she’s got to use it, like I’m sure these things where things are dead simple to use, there’s not a lot involved in them, the odd switch on, switch off, no, it’s too much for her to take in. (Carer; C107).*

Notwithstanding carers frequently persuaded those they cared for to use a device by saying that it was to help the carer look after them.

*She said she felt, it (a pendant alarm) made her feel like a crock, you know (laughing). She says, “I don’t need this, I’m perfectly alright.” And the way that I persuaded her to wear it was, I said, “It just makes me feel better to know that you can contact somebody if you have a fall in the house, or if you’re not too well and you can’t get to the phone.” So, I said “You might not want to wear it, but wear it for me please because it, it stops me worrying about you.” Erm, so that was why she wore it, really. (Carer; C113).*

However, whilst accepting such explanations, some people with dementia routinely judged formal AT as an inconvenience to be tolerated for the sake of their carers, rather than as a benefit to themselves. One person with dementia accepted a range of potentially intrusive DIY technologies including CCTV cameras set up by her husband because she felt it made his life easier.

*Person with dementia (P107): Oh yes. He’s terrified. He lost me in the town once, that was the day when I went to (name of a shop) and asked them if they would look after me bag. He, aye, he was terrified.*

*Interviewer: So you’ve got the cameras, so that he can see that you’re in the house? So he knows that you’re safe?*

*Person with dementia (P107): Yeah.*

Another person with dementia accepted the use of a pendant alarm because she felt it helped her husband and main carer to cope with caring for her, rather than because of its direct benefits;

*Interviewer: How you feel about that, being watched via the pendant?*

*Person with Dementia (P108): Ah, it’s his way of coping with me. He cannot cope with the idea of me having dementia. He cannot cope.*

In a few cases, people with dementia could not be persuaded to use AT or actively resisted its use. Forgetting or not knowing what a device was, several refused to use them particularly devices such as pendant alarms or fall sensors which had to be continuously worn. Reasons given included disliking wearing the device, feeling a technology was unnecessary in their case, because they felt devices would be seen as watching the person with dementia or controlling their actions, or because the person with dementia found devices to be frightening;

*it’s very rare she’ll put the pendant on, you know, and it annoys me because she’s terrified in case, she’s worn it and the time she’s worn it, she’s bent over or knocked it and it’s went off and it’s frightened her. So she’s frightened to put it on, she’ll say she’s frightened to put it on in case it goes off. (Carer; C104)*

In contrast, carers blamed such refusals on personal attributes such as ‘stubbornness’ or ‘laziness’;

*I think certainly think something like the pendant alarm, that’s such a basic thing. But, as it turned it’s no use whatsoever to us but that was just because my mam is such a stubborn individual. (Carer; C113).*

There was frequently a mismatch between what carer thought people with dementia would be able to accomplish and the person’s with dementia actual capabilities in relation to learning how to use new technologies. As a result carers could misinterpret the motives of the person with dementia as they struggled with well-meaning attempts to introduce new technologies, including AT;

*I bring something down, put it on that table and she just won’t touch it. I bought all sorts to try and help her; she’s just not interested. Buy something new and she’s not interested. As I say, I used to think she was just being bloody lazy, but that’s been explained to me that’s not the case. (Carer; C107).*

The routine use of AT in practice therefore afforded the greatest advantages to carers with often only indirect benefits for people with dementia themselves. Such benefits had to be weighed against the potential inconvenience AT could cause and how accepting or tolerant the person with dementia would be of their ‘distress’.

### How is AT integrated into the everyday lives of people with dementia?

#### i) The pivotal role of the family carer

Family carers played a key role in facilitating the integration of AT into the usual routines of their relatives by undertaking much of the everyday work required to ensure their habitual use. Some carers were acutely aware that a person with dementia could potentially learn to use new objects but that they also needed a great deal of continuous assistance to do so.

*You would have to leave an idiot’s guide pinned to the board, explaining to her what it was. And she would read it, then she would forget about it, and possibly over time something would stick, not very much but something might stick, but it’s not guaranteed to stick. (Carer; C123).*

In practice, carers often had to support a person to use often even simple technologies, for example routinely telling them to look at a clock instead of simply giving them the time, or reassuring a person whenever telecare alarms were triggered. This ‘background’ work also had to be continual. While appearing to be simple to use, many devices required a number of tasks to be completed in order to function; for example ensuring a device was charged, or putting it in the same place each day. Carer 109 described the daily activities needed to support her mother in using the ‘Buddi’; a simple handheld device combining a pendant alarm, fall detector, GPS location monitor and activity monitor connected to a telephone monitoring service (www.buddi.co.uk);

*So it became a routine of me just charging (the buddi;) up every evening and then first thing, when she comes downstairs into the sitting room, it’s always on that little placemat on the unit, I leave it in the same place. I don’t have to tell her to pick it up, even though she’s got a really bad memory now she goes straight there and puts it in her handbag. And because she understood at that particular point, it’s become a real part of her routine, even though her memory’s getting worse she still does the same thing ‘cause it’s in her routine, it’s what she does. (Carer; C109).*

If this background work was thoroughly completed by a carer, AT use almost became ‘invisible’ for the person with dementia, with the ultimate aim of reframing the technology as one of the range of routine objects to be used in the home. Nevertheless this work often comprised a wide range of supportive tasks, carried out over a long period of time, to achieve habitual use and thus maximise integration. The routines supporting AT use could also quickly change as a person’s dementia progressed, meaning carers ‘work’ in relation to AT frequently had to adapt and change if AT use was to be sustained.

#### ii) The role of professional carers: too little too late?

In the UK, health and social care services are meant to play an important role in proving information about AT and supporting its use in practice; however, most family carers received little practical support through these formal care services. Formal AT provision was rarely proactive, instead usually occurring as a consequence of a ‘crisis’, such as a fall, or due to ‘luck’, for example chance encounters with health and social care professionals, attending local events in which AT services happened to be present, or carers happening to read about devices in local media or as part of local pilot studies;

*Interviewer: Have you ever come across anything like, you know, like these before?*

*Person with dementia (P101): We went to a conference thing. We didn’t actually look into it but, I’m sure they had stuff like this (AT) there.*

*(PwD) used to always get me a ‘Daily Express’. Just by chance, that very same day was a little article about the Buddi. And I read that article and I thought, “Oh that sounds great.” Anyway, when (PwD) got her diagnosis the consultant mentioned it. And straight away I knew what he was talking about ‘cause I’d read that article. So he said, “Oh, that might be useful for your mam” (…) So she was put on the pilot scheme for it. (Carer; C109).*

Where AT were provided by social care services as part of a formal care package, participants frequently expressed dissatisfaction with both the quantity and quality of support they received once technology had been delivered or installed;

*Interviewer: The box (telecare base unit), and the sensors, have they been helpful at all?*

*Person with dementia (P108): Oh what, since they put it in? Haven’t seen them since. No. I suppose they put it in, can ring the bell if you want it. They haven’t really, I’ve had a letter from them to say they’re reviewed every year, or something. Haven’t seem ‘em since.*

People with dementia and their carers felt that the AT was ‘dropped in’ to their lives with little ongoing information and advice about how to use it; carers thus devised their own ways of making technologies work in practice. Several carers used DIY AT instead of formal AT products partly because they did not know formal AT devices were available but also because they felt commercially available devices, being more familiar, could be more easily adapted to their needs and were better value for money.

*I mean to be honest I actually toyed with the idea of just buying her (PwD) an android phone because you can get them very cheaply these days. Maybe it’s just better to buy something that can you know already. (Carer; C121).*

Family carers who tried to seek information from health and social services about AT reported minimal advice regarding what AT was available and also about how and when AT could be used to help with care. Several also felt frustrated about formal AT being introduced at too late a stage to help, especially if the technology was introduced following a crisis.

*Certainly this assistive technology was something that by that time I almost think that, you know, they introduced it too late, and I didn’t know what the hell it was anyway. (Carer; C114).*

Timing was crucial. Both carers and the person with dementia needed time to familiarise themselves with AT, but both felt that unless the AT was introduced early in the disease trajectory, family carers could not provide the essential support to enable their relative to learn to use the technology effectively. However paradoxically a number of carers were also resistant to the idea of introducing AT ‘too early’, i.e. before they felt it was necessary. While early installation could support a person to learn a device, it could also be ‘institutionalising’, for example disrupting the meanings attached to a person’s home, or even forcing a person to engage with the fact that they were ‘ill’, which could be distressing. One carer questioned the appropriateness of dementia friendly signs in the home:

*When your memory isn’t too bad then my gut reaction is it’s institutionalising. It’s like, I know where the toilet’s at, I know where our bedroom’s at. And if it gets so bad where I don’t know where these things are at, then then seeing “Dining room” wouldn’t make much sense to me. (Carer; C108).*

When to introduce AT therefore became a key dilemma for family carers when making decisions about AT use, but an area in which they largely felt unsupported in their routine contact with formal care services.

### What issues influence the use of AT among people with dementia and their carers?

Carers were generally positive about using AT. They cited benefits as providing support with caring tasks, giving them ‘peace of mind’ and offering greater independence for the person with dementia. One carer claimed that using the ‘Buddi’ system had enabled her to continue working;

*Interviewer: How have you felt about using the Buddi?*

*Carer (C109): Tremendous. As I say, I couldn’t have continued working as long as I did, and I’m still, we’re still benefitting from it you know. It, it’s really, I think it’s a wonderful device, wonderful.*

However other carers also commented that they felt AT was a ‘necessary evil’; beneficial in terms of helping with care, but less preferable to care provided by a ‘real person’. Indeed AT could be judged as superfluous, or even with hostility, if it was seen as a replacement for personal care, for example if the technology took over completely the carer’s own caring duties or replaced a task carers felt they could do better;

*I mean, that (a pill dispenser)’s brilliant if you’re on your own. But you don’t need it as a couple. I mean, I put his tablets out now on a morning. He still takes his own on a night. (Carer; C112).*

In contrast people with dementia were often ambivalent about using AT. As noted above, several people with dementia refused to use AT, or used it to please their carers. In addition, AT use by people with dementia was frequently subtly, or sometimes significantly, different from their original intended purpose. Specifically, many people with dementia adapted their use of AT in order to make them better fit into their lives;

*I have a pendant. I don’t wear it when he’s in the house ‘cause he’s always near me, you know. But I wouldn’t be without that pendant, just in case. ‘Cause I’ve fallen at night you see, and he’s a very heavy sleeper, so we keep it, I keep it right beside me at night. (Person with dementia; P108).*

Practical difficulties in using AT could present barriers to their acceptance. Telecare or pendant alarms were designed to be passive, only alerting a person to their presence in the event of an emergency. However in practice their activation occurred much more frequently, with potentially distressing consequences. In particular, the accidental triggering of telecare alarms, particularly when they happened more frequently than expected, could leave a person with dementia fearful of the technology;

*Interviewer: Do you wear the (pendant alarm)?*

*Person with dementia (P104): I’ll tell you the truth, I’m terrified of it. When we first got it it was over there and I didn’t know what it was, and I happened to go over and I touched it and I thought, “What is it?” Of course the voice came up straight away, “What’s the matter?” It must go to a centre, some centre. And I said, “Oh I’m sorry, I must’ve touched something”.*

One person with dementia no longer went into her kitchen after dark because the continual triggering of her telecare system’s door exit alarm, activating when near the external kitchen door, frightened her. The ‘disembodied’ voices speaking through telecare intercoms due to emergency callouts, accidental alerts or routine maintenance also caused distress;

*Even the one I’ve got now, you can, cameras you can talk to them. You can imagine a voice coming from there, so that’s not of any benefit really. You’re not reassuring her at all, you’re scaring the daylights out of her. (Carer; C106).*

Yet in several cases carers, nonetheless remained happy for telecare alarms to remain in place despite their distress, largely because it reassured them as to the safety of the person they cared for;

*Interviewer: how do you feel about (mother/P104) being frightened of touching the door?*

*Carer (C104): It doesn’t worry us, because I know if she doesn’t touch the door she’s not going to attempt to go out.*

Such issues raise questions about whether the perceived benefits of some AT for carers and for formal services outweigh either the inconvenience or emotional distress experienced by the person with dementia, and ultimately who AT is being installed and used for.

### Cost as a barrier to AT acceptance

Both the direct and indirect costs of AT were repeatedly highlighted as a potential barrier to their use. Several carers noted that AT was generally expensive.

*You know, some people can’t afford it. I don’t mind paying for it ‘cause it’s helping her (mother) but I think it, it is expensive. It is quite steep, but then again, if her attendance money is there for it and she needs it, you, you don’t mind getting it if it’s going to help her, you know. (Carer; C104).*

Charging models to rent AT varied across geographical localities. Formal AT was rented via local authority community alarm or telecare services, although some people receiving certain state benefits could secure them without charge. While AT were considered expensive, a few carers and people with dementia said they were willing to pay if they could see the advantages of using them or if they were deemed to be worth the cost relative to other products;

*But I, I have, well I, I’ve always had the same thing, always buy the best you can afford So, for example, if you can get something for £20, but if you can afford £100, get something and then it’s going to last for years, you know, and it’s not going to break in six months. (Person with Dementia; P101)*

While many considered local authority service charges acceptable, some felt rental charges of around £20 per month were too expensive, particularly for people living on limited incomes or paying for other social care services.

*Because I, we can’t keep the heating, can’t keep, we can only just about keep this heating on. We just can’t afford to survive on our money. (…) If I had to pay for it (a telecare system) it would have to go. (Person with dementia; P108).*

In addition, despite being sold as AT, ‘off the shelf’ AT products were rarely available through formal AT services who in general only supplied ‘telecare’. Instead such products had to be purchased either through the private AT market or by buying their commercial, ‘do it yourself’ equivalents. Yet even among those who were willing to consider AT, the perceived increases in cost when compared to what were judged as simple or mature technologies were re-labelled as ‘assistive’ or ‘medical’ technologies were also identified as barriers,

*As soon as it’s got a tag on it, like a dementia or disabled or something like that, it suddenly becomes ten times more expensive. I can’t see why that would cost so much when, you know, it’s not massive technology this. It’s not rocket science. (Person with dementia; P101).*

Perceptions regarding high formal AT costs, particularly when compared to generic household products which fulfilled similar functions therefore posed a key barrier both for the general acceptability of AT products, and for the continuing development of a mixed economy of AT provision in dementia.

## Discussion

This study explored the everyday use of technology by people with dementia and their family carers (current and former), the types and range of devices they used and how they integrated the technology into their usual routines, including key factors which influenced this. To date, how people with dementia and their families use technology in their everyday lives has received scant empirical consideration [7, 28]. In terms of attitudes to AT use, people with dementia appear generally positive especially if it can facilitate autonomy and independence [21]. Our findings also showed that in real world scenarios, when AT is instigated in an individual, person-centred way, it has the potential to positively influence the lives of families living with dementia [7, 31]. Importantly no-one in our study avoided the use of technology per se; technology use depended upon individual reasons and individual contexts, with perceptions of usefulness rather than need being the biggest driver of uptake [28]. Notwithstanding it was family carers who appeared to receive greatest benefit through maintaining the person with dementia’s safety, and minimising their anxiety, rather than facilitating their participation in everyday activities. Such a risk adverse approach by family carers of people with dementia has often been demonstrated and is not surprising if they are relatively unsupported by formal care services [20]. Godwin [32] developed an ethical checklist for professionals assessing the possible use of AT in dementia which may help to address the complexity of needs experienced by both carers and people with dementia. A care manager or dementia advisor, who can act as an advocate for the person with dementia and whose agenda is focused on increasing social inclusion, may be the missing link.

A mixed economic provision of AT was found with our participants using three categories of technology: formal, accessed via health and social care services; ‘off the shelf’ devices purchased directly from the private sector and ‘DIY’, whereby carers adapted or refined everyday devices or systems to meet their individual needs. The main benefit of AT to families living with dementia was to support carers in their caring role, with people with dementia sometimes tolerating personal inconvenience to facilitate this. Family carers played a pivotal role in enabling AT integration into a person with dementia’s everyday routine, with responsibility for product identification, and installation and ongoing management, through understanding and undertaking the physical and emotional work needed to support the person with dementia to use the technology. Barriers to AT use included: perceptions about the high cost of formal AT; dilemmas about timing and at what stage to use AT; and a lack of information and support from formal health and social care services about how to access AT, where to source it and when and how it can be used.

To achieve successful AT integration into their daily lives, family carers had to undertake considerable work as the main gatekeeper and decision maker, to become the ultimate beneficiaries of AT use [33, 34]. Nevertheless they experienced numerous challenges. The experience of seeking information about, and accessing and using, AT appeared to be a chaotic and confusing process, dependent on carers who were knowledgeable and confident enough to negotiate the multiple information routes available, often with little to no formal assistance from formal agencies. Considerable carer input was also required to ensure full integration of AT into their usual care routines; this was also largely unsupported by the wider formal care system. In the UK, the lack of support to facilitate access to AT and its everyday use, alongside perceptions regarding high costs of the technology, means that the mixed economy approach to AT in dementia in its current form arguably fails many of those in greatest need.

Providing individualised support which ensures full integration of technology within a person’s routines has been acknowledged as a key driver of technology use in dementia [7, 28]. Recent findings from another UK study noted that standardisation in both the design and delivery of general AT platforms failed to account for individual’s lived experiences, leading users to reconfigure technologies in order to meet individual need [18, 19]. In addition, the lack of a nuanced understanding of effects of cognitive impairment among formal and informal carers could make standardised telecare platforms inappropriate. Such experiences were also common in our study. These problems are further exacerbated when AT use is unsupported by formal health and social care services, potentially leading to the abandonment of AT. Our findings show that formal care services became a barrier, rather than a facilitator, to AT use by forcing families, by their absence, to find AT devices and/or systems themselves and failing to offer post supply support, with carers often feeling abandoned, after devices were installed. Understanding how AT use becomes part of the mundane activities of an individual’s daily life and how this can be most effectively facilitated by formal health and social care services is one of the key drivers of future AT implementation [18, 28].

This study addressed a neglected area of research investigating how AT is routinely used in the daily routines of people with dementia and their family carers, and the challenges they face in accessing both information about AT and how best to use it. However the research was limited to one geographical area in one area of the UK, so our findings may have limited generalisability to other settings. In particular different localities within the UK, and also other countries, will have different levels of statutory provision of AT and also more varied access to the private sector, which may shape access to AT at local, regional and national levels [6].

## Conclusion

It has been argued that the ‘mixed economy’ landscape, with private AT provision supplementing state provision of AT, is a key feature for the mainstreaming of AT services [14]. Our data suggests that such a mixed economy is indeed taking place, with more participants using ‘off the shelf’ and ‘DIY’ AT purchased privately rather than via health and social care services. Most of these products were scaled down, commercial versions of existing household technologies, simple adaptations carried out by individual family carers, or more complex IT installations set up by knowledge-able carers. However this system has largely arisen due to an inability of formal care services to meet client needs. Such findings therefore raise questions about just who AT in its current provision is working for [35] and whether a mixed market approach is the most appropriate provider model. In the UK, patient and family carers still consider formal health and social care providers to have a key role in this area of dementia care, but levels of professional knowledge and service support appear currently far from adequate to meet need. Our findings suggest that everyday technologies play an important role in supporting families with dementia to continue caring; determining how everyday technologies can therefore be used in conjunction with AT within effective and person-centred models for future AT provision is therefore a subject for both further research, and for future service development.

## References

[1] Prince M, Albanese E, Guerchet M, Prina M, Prince M, Albanese E, et al. World Alzheimer Report 2014. Dementia and risk reduction: an analysis of protective and modifiable risk factors. London: Alzheimer's Disease International; 2014.

[2] Prince M, Knapp M, Guerchet M, McCrone P, Prina M, Comas-Herrera A (2014). Dementia UK: Second Edition.

[3] Prince M, Prina M, Guerchet M (2013). World Alzheimer Report 2013. An analysis of long term care for dementia.

[4] Duff P, Dolphin C (2007). Cost-benefit analysis of assistive technology to support independence for people with dementia – part 2. Results from employing the ENABLE cost-benefit model in practice. Technol Disabil.

[5] Knapp M, Iemmi V, Romeo R (2013). Dementia care costs and outcomes: A systematic review. Int J Geriatr Psychiatry.

[6] Gibson G, Newton L, Pritchard G, Finch T, Brittain K (2014). Robinson L.

[7] Astell A, Malone B, Williams G, Hwang F, Ellis M (2014). Leveraging everyday technology for people living with dementia: a case study. J Assist Technol.

[8] Fry G, Buse, C. The benefits of telecare for service users and carers: existing empirical evidence. In Aktive Consortium. The role of telecare in meeting the care needs of older people. Aktive research report vol 1. 2013. Retrieved from http://www.aktive.org.uk/downloads/AKTIVE_Report_Vol_1_16.05.pdf.

[9] Davies A, Rixon L, Newman S (2013). Systematic review of the effects of telecare provided for a person with social care needs on outcomes for their informal carers. Health Soc Care Community.

[10] Steventon A, Bardsley M, Billings J, Dixon J, Doll H, Beynon M, et al. Effective of telecare on use of health and social care services: findings from the Whole Systems Demonstrator Cluster Randomised trial. Age Ageing. 2013;42:501–8. 10.1093/ageing/aft008 PMC3684109 23443509

[11] Fleming R, Sum S (2014). Empirical studies on the effectiveness of assistive technology in the care of people with dementia: a systematic review. J Assist Technol.

[12] Brittain H, Corner L, Robinson L, Bond J (2010). Ageing in place and technologies of place: the lived experience of people with dementia in changing social, physical and technological environments. Sociol Health Illn.

[13] Department of Health (2012). Prime minister’s challenge on dementia.

[14] Barlow J, Curry R, Chrysanthaki T, Hendy J, Taher N (2012). Remote care PLC: Developing the capacity of the remote care industry to supply Britain’s future needs.

[15] Woolham J, Gibson G, Clarke P (2006). Assistive technology, telecare and dementia: Some implications of current policies and guidance. Res Policy Plan.

[16] Burrow S, Brooks D (2012). ATDementia: an information resource on assistive technologies that help support the independence of people with dementia. Dementia.

[17] Telecare Aware. O2 to stop selling telecare and telehealth in the UK. 2013. Retrieved from http://telecareaware.com/o2-to-stop-selling-telecare-telehealth-in-the-uk/.

[18] Greenhalgh T, Wherton J, Sugarhood P, Hinder S, Procter R (2013). What matters to older people with assisted living needs? A phenomenological analysis of the use and non-use of telehealth and telecare. Soc Sci Med.

[19] Procter R, Greenhalgh T, Wherton J, Sugarhood P, Rouncefield M, Hinder S (2014). The day to day co-production of ageing in place. Comput Supported Co-operative Work.

[20] Robinson L, Hutchings D, Corner L, Finch T, Hughes J, Brittain K, et al. Balancing rights and risks: Conflicting perspectives in the management of wandering in dementia. Health Risk Soc. 2007;9:389–406.

[21] Robinson L, Brittain K, Stephen L, Jackson D, Olivier P (2009). Keeping In Touch Everyday (KITE) project: developing assistive technologies with people with dementia and their carers to promote independence. Int Psychogeriatr.

[22] Hughes R (2009). “safer walking” issues and ethics in the use of electronic surveillance of people with dementia. J Assist Technol.

[23] Zwijsen S, Neimeijer A, Hertogh C (2011). Ethics of using assistive technology in the care for community-dwelling elderly people: an overview of the literature. Aging Ment Health.

[24] Landau R, Werner S (2012). Ethical aspects of using GPS for tracking people with dementia: recommendations for practice. Int Psychogeriatr.

[25] Bowes A, Dawson A, Greasley-Adams C. Literature Review: the cost effectiveness of assistive technology in supporting people with dementia. 2013. Retrieved from http://dementia.stir.ac.uk/system/files/filedepot/1/the_cost_effectiveness_of_assistive_technology_in_supporting_people_with_dementia_october_13_1.pdf.

[26] Department of Health (2013). Research and development work relating to assistive technology 2012–2013.

[27] Leroi I, Woolham J, Gathercole R, Howard R, Dunk B, Fox C, et al. Does telecare prolong community living in dementia? A study protocol for a pragmatic, randomised controlled trial. Trials. 2013;14:gib349. doi:10.1186/1745-6215-14-349. 10.1186/1745-6215-14-349 PMC4015839 24152600

[28] Nygard L (2008). The meaning of everyday technology for people with dementia who live alone. Dementia.

[29] Robinson L, Gibson G, Kingston A, Newton L, Pritchard G, Finch T, et al. Assistive technologies in caring for the oldest old: A review of current practice and future directions. Aging Health. 2013;9:365–75.

[30] Glaser B (1965). The Constant Comparative method of qualitative analysis. Soc Probl.

[31] Arntzen C, Holthe T (2014). Jentoft R.

[32] Godwin B (2012). “The ethical evaluation of assistive technology for practitioners: a checklist arising from a participatory study with people with dementia, family and professionals. J Assist Technol.

[33] Rosenberg L, Kottorp A, Nygard L (2012). Readiness for technology use with people with dementia: the perspectives of significant others. J Appl Gerontol.

[34] Rosenberg L, Nygard L (2012). Persons with dementia become users of assistive technology: a study of the process. Dementia.

[35] Greenhalgh T, Procter R, Wherton J, Sugarhood P, Shaw S (2012). The organising vision for telehealth and telecare: discourse analysis. BMJ Open.

